# Histological Evaluation of Corneal Scar Formation in Pseudophakic Bullous Keratopathy

**DOI:** 10.1371/journal.pone.0039201

**Published:** 2012-06-14

**Authors:** Ting Liu, Yuanyuan Xu, Dapeng Sun, Lixin Xie

**Affiliations:** State Key Laboratory Cultivation Base, Shandong Provincial Key Laboratory of Ophthalmology, Shandong Eye Institute, Shandong Academy of Medical Sciences, Qingdao, China; University of Reading, United Kingdom

## Abstract

**Purpose:**

To evaluate histological changes in the corneal stroma in pseudophakic bullous keratopathy.

**Methods:**

Twenty-eight patients (28 eyes) with pseudophakic bullous keratopathy underwent therapeutic penetrating keratoplasty at Shandong Eye Institute between January 2006 and November 2011. The patients were divided into two groups according to the duration of bullous keratopathy (<1.0 year group or >1.0 year group), and three buttons from enucleated eyes with choroidal melanoma served as a control. In vivo confocal microscopy examination, hematoxylin–eosin, Masson's trichrome stain and Van Gieson staining were used for microscopic examination. The histological evaluation and scoring of the buttons for morphological changes, including the degree of stromal scars, neovascularization and inflammatory cells within the corneal buttons, were compared. To study the underlying mechanism, connective tissue growth factor (CTGF) and TGF-β immunohistochemistry were performed.

**Results:**

Confocal microscopy examination and histological evaluation and scoring of the buttons showed that compared with the <1.0 year group**,** stromal scars, neovascularization and inflammatory cells were more severe in the >1.0 year group (P<0.05). There was an increase in CTGF- and TGF-β1-positive stromal cells in the >1.0 year group.

**Conclusions:**

During the progression of pseudophakic bullous keratopathy, stromal scars occurred more often in the patients that had a longer duration of disease. Cytokines such as CTGF and TGF-β1 may play a role in this pathological process and deserve further investigation.

## Introduction

Pseudophakic bullous keratopathy has recently emerged as a leading cause of endothelial keratoplasty (EK). EK maintains most of the corneal structure and the integrity of the eye as a result of a small incision, no sutures and rapid recovery; therefore, it is increasingly becoming the preferred method for treating bullous keratopathy [1]–[3]. However, not all of the patients with bullous keratopathy can benefit from endothelium transplantation. Particularly in patients with a long duration of bullous keratopathy, EK cannot produce good vision because of the intense corneal stromal scars and neovascularization; thus, penetrating keratoplasty should be used [4]–[6].

During the development of pseudophakic bullous keratopathy, there are pathological changes in the corneal stroma, such as scar formation, the proliferation of a collagenous layer and fibrillar material deposits. An abnormal proliferation of the posterior collagenous layer and a significantly lower keratocyte density was found in the posterior part of the stroma of patients with long-term pseudophakic bullous keratopathy [7]. It has also been reported that abnormal fibrillar materials posterior to Descemet's membrane and subepithelial fibrocellular materials disrupt the epithelial basement membrane and Bowman's layer in the corneas of bullous keratopathy patients [8].

These studies suggested that pathological changes occurred in the corneal stroma during pseudophakic bullous keratopathy. However, the relationship between corneal scar severity and the progression of bullous keratopathy is still unclear. In addition, the underlying molecular mechanism is unknown. Recent studies have shown that CTGF promotes corneal scar formation, and CTGF expression significantly increases during corneal wound healing [9]. CTGF was also found to be involved in the TGF-β1-mediated stimulation of myofibroblast differentiation and collagen matrix contraction in the presence of mechanical stress [10].

However, whether CTGF plays a role during scar formation in bullous keratopathy has not been reported. This study may provide a histological and molecular reference for the evaluation of corneal scar formation during the progression of pseudophakic bullous keratopathy.

## Materials and Methods

This retrospective institutionally reviewed study was approved by the Institutional Review Board of Shandong Eye Institute and conducted in accordance with the ethical standards of the 1964 Declaration of Helsinki. We obtained written informed consent from all participants involved in our study. The series consisted of 28 patients (13 females and 15 males) with a clinical diagnosis of different durations of pseudophakic bullous keratopathy at Shandong Eye Institute between January 2005 and November 2011. All of the patients presented eye pain, corneal edema, and an obvious low endothelium count (from less than 600 cells/mm to none detectable).

Based on the duration of pseudophakic bullous keratopathy, the patients were divided into two groups: the <1.0 year group with 9 patients (4 males, mean duration of 0.5±0.2 years, and mean age of 50.7±14.5 years) and the >1.0 year group with 19 patients (11 males, mean duration of 3.1±2.9 years, and mean age of 58.3±15.1 years). Three buttons from enucleated eyes with choroidal melanoma served as the control.

### In Vivo Scanning Confocal Microscopy

Confocal microscopy images were obtained before surgery. Informed consent was obtained from all of the patients before the examination. After one drop of 0.5% proparacaine hydrochloride (Alcon Laboratories, Fort Worth, Texas, USA), the central cornea was examined with a scanning confocal microscope (Confoscan 4; Nidek Technologies America, Greensboro, North Carolina, USA). The center and four quadrant points within 6 mm of the central cornea were examined. Serial images were acquired with a 403 non-applanating immersion lens that had a concave surface and a working distance of 1.9 mm. Approximately 300 sequential images were obtained from the endothelium to the epithelium during a single examination.

### Corneal Staining

Details regarding penetrating keratoplasty have been reported previously [11]. All of the surgeries were performed under general anesthesia. The corneal buttons were half-cut along the central line. Serially graded ethanol baths followed by xylene were used to dehydrate the tissues before they were immersed in paraffin wax. The samples were embedded in paraffin molds, sectioned at 4-µm thickness, and mounted on glass slides. To determine whether the central corneal buttons was representative, serial 4-µm sections were cut through the entire central cornea at 200-µm intervals. Because the slides were found to be consistent from section to section, representative sections from the central corneal buttons were used. Hematoxylin–eosin, Masson's trichrome stain and Van Gieson stain were used for microscopic examination and evaluation.

### Histological Evaluation and the Scoring of the Buttons

The buttons were histologically evaluated and scored, including the thickness of the stromal scars, the number of vessels in the neovascularization and the number of inflammatory cells within corneal buttons. The pathological changes were graded from 0 to 3 based on the severity of each characteristic. For stromal scar thickness, a thickness of the stromal scars less than 1/3 of the entire stroma was graded as 1, 1/3-2/3 of the entire stroma was graded as 2, greater than 2/3 of the entire stroma was graded as 3, and no scars was graded as 0. Six high-power fields were randomly chosen in each slide, and the number of vessels and the number of inflammatory cells per high-power field were counted within corneal buttons. All of the slides were evaluated by the same pathologist. The specimens were coded on the posterior surfaces, and the diagnosis was unknown to the observer. The total score and each characteristic were statistically analyzed. For each cornea, measurements were obtained for every parameter, and the means of the values were calculated.

### Immunohistochemistry

For immunohistochemistry, 4-µm sections (repeated at least three times for each group) were obtained from the paraffin-embedded corneal buttons. The antigens were recovered by microwaving for 15 min in EDTA solution. Endogenous peroxidase activity was quenched by incubating the sections in 3% hydrogen peroxide for 5 min. Normal goat serum was used to block nonspecific staining. Subsequently, the sections were incubated with mouse anti-TGF-β1 (Maxin, Fujian, China) or rabbit anti-CTGF (Abcam, Hong Kong) for 60 min at 37°C, a reinforcing agent (Maxim, Fujian, China) for 15 min at 37°C, and HRP conjugated goat anti-rabbit IgG for 30 min at 37°C. Peroxidase activity was visualized by incubating the sections in diaminobenzidine (DAB) solution (Maxim, Fujian, China). Negative controls were performed in the absence of primary antibodies. Finally, the samples were mounted and examined with a microscope (Nikon Eclipse E800, Nikon, Tokyo, Japan).

### Statistical Analysis

Significant differences among the three groups were evaluated with the Student-Newman-Keuls one-way ANOVA using the SPSS 13.0 software. The mean ± standard deviation is shown, and *P*-values <0.05 were considered statistically significant.

## Results

### Clinical Treatment

No infection was observed after surgery, and no postoperative complications occurred during follow-up. The best ocular vision was significantly increased after penetrating keratoplasty, and the intraocular pressure remained stable during the follow-up period.

### Clinical Examination with in vivo Scanning Confocal Microscopy

**Figure 1:**
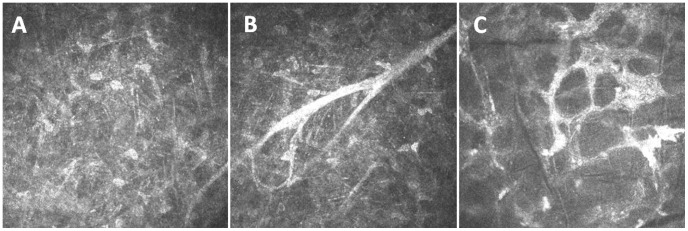
Representative images of stromal scars and blood vessels in the corneal stroma of the more than 1.0 year group. (A) The dense, highly reflective, tiny needle-shaped materials in the deep stroma; (B) obvious neovascularization in activated stromal cells; and (C) the region of corneal scars showed cystic corneal edema and a honeycomb structure, and the cell nucleus was rarely observed.

Using an in vivo scanning confocal microscope, there was no obvious scar formation, neovascularization or inflammatory cell infiltration present in the <1.0 year group or the control group. However, in the >1.0 year group (disease duration of 3–4 years), there were dense corneal scars, obvious neovascularization, activated stromal cells and inflammatory cells. Dense, highly reflective, tiny needle-shaped materials were present in the deep stroma. Cystic corneal edema and a honeycomb structure were found in the area containing corneal scars (Figure 1).

### Histological Evaluation and Scoring of the Buttons

**Figure 2:**
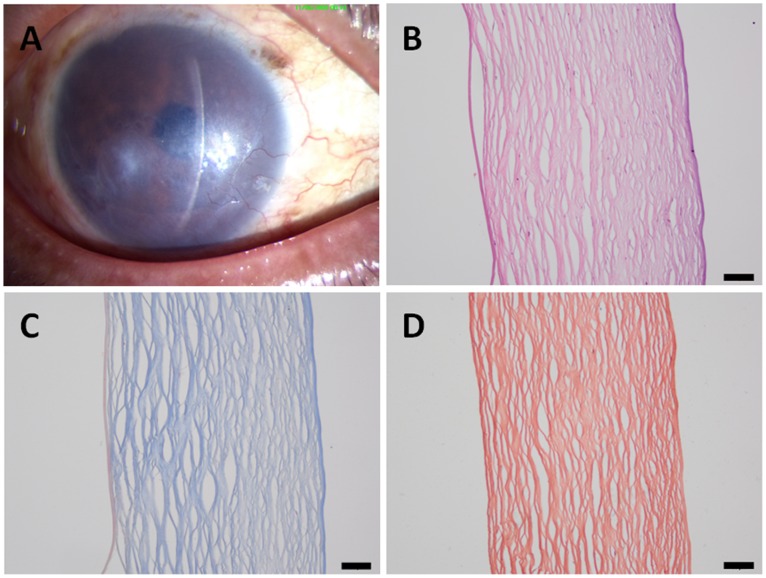
A patient with a 0.5-year history of pseudophakic bullous keratopathy. (A) A slit-lamp photograph of the cornea showing no obvious scar formation; (B) in the histological sections, no obvious scars, neovascularization or inflammatory cells can be observed; (C) Masson's trichrome staining; and (D) Van Gieson staining. **Scale bar 50 µm.**

**Figure 3:**
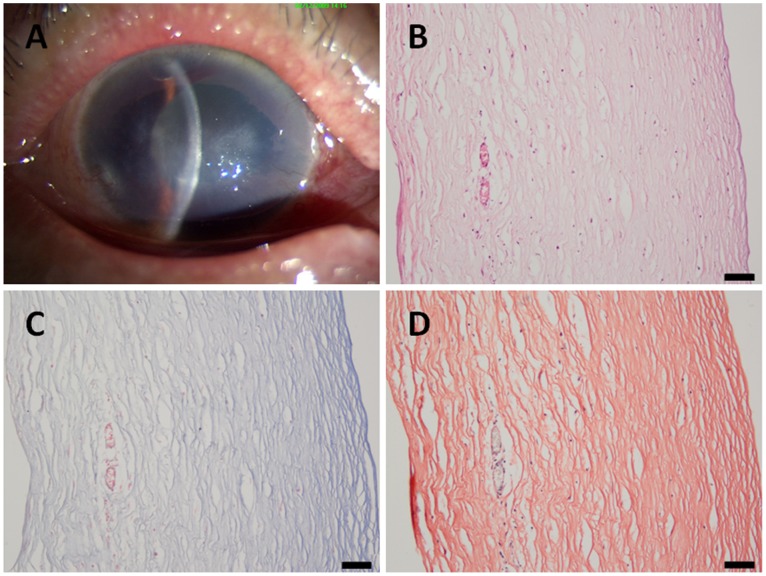
A patient with a 1.5-year history of pseudophakic bullous keratopathy. (A) A slit-lamp photograph of the cornea showing a mild scar and neovascularization; (B) in the histological sections, scars, neovascularization and inflammatory cells can be observed; (C) Masson's trichrome staining; and (D) Van Gieson staining. **Scale bar 50 µm.**

**Figure 4:**
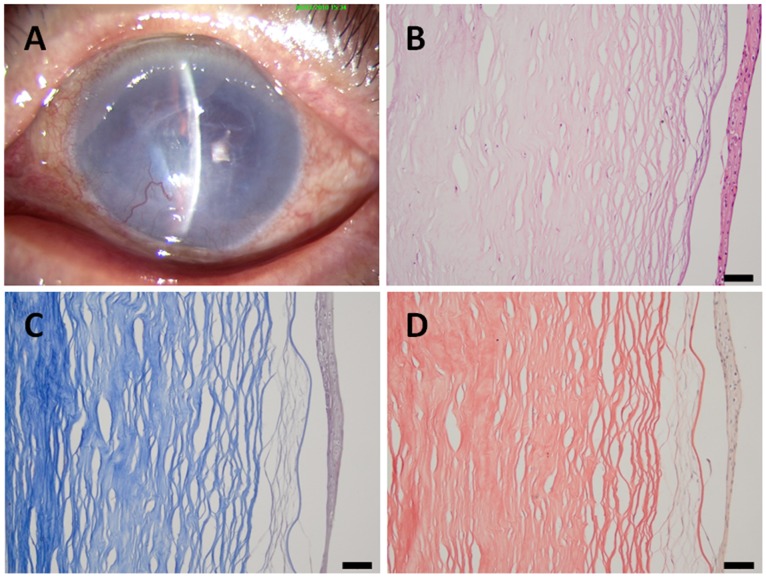
A patient with a 3-year history of pseudophakic bullous keratopathy. (A) A slit-lamp photograph of the cornea showing dense scarring and neovascularization; (B) in the histological sections, obvious intense scarring, neovascularization and inflammatory cells can be observed; (C) Masson's trichrome staining; and (D) Van Gieson staining. **Scale bar 50 µm.**

In the <1.0 year group, corneal stromal fiber crosslinking was found in the deep stroma, and no dense deep corneal stromal scars were found. In addition, neither neovascularization nor inflammatory cell infiltration was found (Figure 2). In the >1.0 year group, the epithelial and stromal pathological changes were more extended and severe. There were dense corneal scars, neovascularization and inflammatory cell infiltration in the stroma. Anterior corneal scars and broken Bowman’s membranes were also observed (Figures 3, 4).

**Figure 5:**
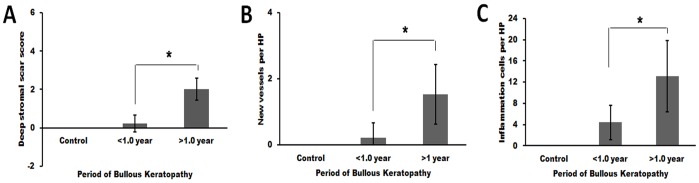
Evaluation of morphological changes in patients with bullous keratopathy. Significant differences were found between the more than 1.0 year group and the less than 1.0 year group (*, P<0.05), including (A) corneal stromal scar grading, (B) the number of new vessels per high-power field, and (C) the number of inflammatory cells per high-power field.

The histological evaluation scores of corneal stromal scars and the number of new vessels and inflammatory cells per high-power field were obviously higher in the >1.0 year group than the <1.0 year group (*P* = 0.00, 0.00, and 0.01, respectively) (Figure 5).

### Immunohistochemistry

**Figure 6:**
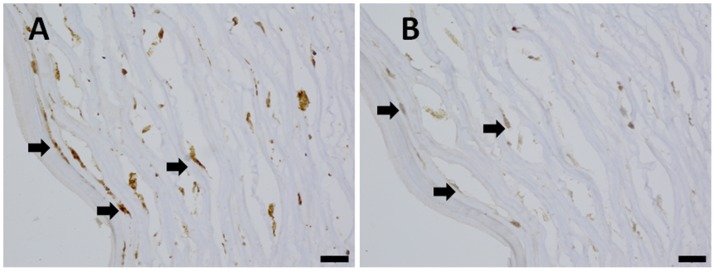
CTGF and TGF-β expression in the more than 1.0 year group. (A) The CTGF expression in the deep stroma (arrow); and (B) the TGF-β expression in the deep stroma (arrow). **Scale bar 20 µm.**

In the corneal scars of the >1.0 year group, many CTGF- and TGF-β1-positive cells were found scattered throughout the deep stroma (Figure 6), and the positive cells were mainly fibroblasts. However, only a few TGF-β1- and CTGF-positive cells were found in the corneal stroma of the <1.0 year group, and no positive cells were found in the control group.

## Discussion

Previous reports of bullous keratopathy have primarily been focused on the endothelial cell loss that constitutes the underlying pathogenic basis of this disorder [12]–[13]. Recent studies have demonstrated that corneal surface and stromal pathological changes occur in patients with long-term bullous keratopathy. Pseudophakic bullous keratopathy has been reported to present an abnormal corneal ocular surface in which superficial cell layers are exfoliated, leaving breaches in the protective MUC16 glycocalyx [14]. Corneal stromal pathological changes in patients with bullous keratopathy have also been demonstrated with in vivo laser confocal microscopy. Highly reflective, tiny needle-shaped materials have also been found in the corneal stroma, and the appearance of these materials may be caused by crystalline or lipofuscin [15], [16]. But the relationship between the clinical disease duration time and the scar formation had not be systemically studied before.

In our study, we also found highly reflective, tiny needle-shaped materials in the corneal stroma of the >1.0 year bullous keratopathy group with confocal microscopy. These needle-shaped materials were not found in the <1.0 year bullous keratopathy group. A prolonged period may be required to form deposits, such as the needle-shaped materials, in the corneal stroma. We also evaluated the scars with Masson’s trichrome and Van Gieson staining to confirm the correlation between the severity of stromal pathological changes and the clinical duration. The score of stromal scars, the number of vessels and inflammatory cells per high-power field all increased in the >1.0 year bullous keratopathy group.

The molecular basis of long-term bullous keratopathy has been investigated in different extracellular matrixes, such as collagen and fibronectin, and with different cytokines, including the transforming growth factors. Fibrillar deposits of an anti-adhesive glycoprotein tenascin in the anterior and posterior stroma, epithelial basement membrane, subepithelial fibrosis areas, and posterior collagenous layer were found in bullous keratopathy corneas [17]. These fibrotic changes may play a significant role in the progression of bullous keratopathy. In corneas with bullous keratopathy, significantly lower transcriptional activity of TGF-beta3 mRNA was also found compared with normal tissues [18]. TGF-β1 had been found in human corneas with the bullous keratopathy, and our findings were consistent with previous studies. However, the current study was the first to find that CTGF, as the effect molecule of TGF-β1 pathway, may play a more directly role during the corneal scar formation, like in the development of human bullous keratopathy. In our study, the TGF-β1-positive cells and CTGF-positive cells were mainly found in the scar area, and the locations of the positive cells were nearly identical in the >1.0 year group. This result indicated that as bullous keratopathy progresses, more inflammation-related or scar formation-related cytokines might appear in the corneal stroma. Recent fundamental studies have demonstrated that CTGF may play a key role in the human corneal scar formation [19]–[21]. Our clinical studies provide a direct clinical samples based proof, and may help to pave a bridge to apply the CTGF target from bench to bedside. There may be a way to use this information to reduce or prevent permanent corneal scarring/fibrosis in the future.

Endothelial keratoplasty has been an alternative to penetrating keratoplasty for the treatment of bullous keratopathy [22]–[26]. To treat a stromal superficial scar of pannus, Yang et al [27] used superficial keratectomy as an initial surgical stage in performing successful endothelial keratoplasty in a severe bullous keratopathy patient and achieved a good visual outcome. This patient mainly had a superficial scar of pannus immediately anterior to Bowman’s membrane, which allowed for superficial keratectomy as an initial surgical procedure for the complete removal of the irregular anterior scarring and pannus. Most importantly, the patient only had mild deep stromal scarring.

In our series, all of the patients in the >1.0 year group acquired a good visual outcome after penetrating keratoplasty. This result indicated that penetrating keratoplasty was a good choice for long-term bullous keratopathy. For mild stromal scarring, a 2-stage procedure can be used to broaden the indication for endothelial keratoplasty. However, our study was only a histological study with limited corneal button samples. Further investigations are needed to help determine stromal scar changes and the molecular mechanism.
